# Toxicity Effects of Toad (*Rhinella jimi* Stevaux, 2002) Venom in Chicken (*Gallus gallus domesticus*)

**DOI:** 10.1155/2014/851473

**Published:** 2014-06-19

**Authors:** Ivana Cristina Nunes Gadelha, Joseney Maia de Lima, Jael Soares Batista, Marilia Martins Melo, Benito Soto-Blanco

**Affiliations:** ^1^Programa de Pós-graduação em Ciência Animal, Universidade Federal Rural do Semi-Árido (UFERSA), BR 110 Km 47, 59628-360 Mossoró, RN, Brazil; ^2^Departamento de Clínica e Cirurgia Veterinárias, Escola de Veterinária, Universidade Federal de Minas Gerais (UFMG), Avenida Antônio Carlos 6627, 31275-013 Belo Horizonte, MG, Brazil

## Abstract

This study aimed to evaluate the pathological changes that occur after administering different doses of *R. jimi* (Stevaux, 2002) parotoid glands secretion to *Gallus gallus domesticus* chicks. Twenty-three animals were used in this study and were divided into 5 groups that received a toad venom dose of 0, 3.0 mg/kg, 6.0 mg/kg, 10.0 mg/kg, and 25.0 mg/kg. After 48 h, the necropsy and pathological examinations were performed. No clinical signs of toxicity were observed in any group. Macroscopically, hepatomegaly, areas of liver necrosis, splenomegaly, necrotic and hemorrhagic cardiac regions, hydropericardium, dark necrotic lesions of Meckel's diverticulum, and hemorrhages in the lungs and kidneys were detected. Histopathological changes included diffuse vacuolar degeneration of hepatocytes, severe sinusoidal congestion, focal areas of hemorrhage in the parenchyma, swollen cardiac fibers, necrotic myocardial fibers, moderate to acute diffuse alveolar hemorrhage, vacuolar degeneration of the renal tubular epithelium, necrosis of renal tubules, and extensive hemorrhagic areas below the brain and cerebellar meninges. In conclusion, pathological changes of the *R. jimi* toxins in chicks were noted in the heart, spleen, liver, Meckel's diverticulum, lungs, and kidneys. Most of the changes were similar to those observed in humans and animals exposed to toxins from other toad species.

## 1. Introduction

The toad genera* Bufo* and* Rhinella*, which belong to the Bufonidae family, possess various granular glands that secrete toxins for protection against predators. One such is the parotoid gland, which is located in the postorbital region, on both sides, and is specialized in toxin production and storage. In the most toxic species, the parotoid glands are well developed, allowing the storage of large quantities of the venom [1–3].

Toad toxin mainly consists of derived steroids (bufadienolides and bufotoxins) and biogenic amines (epinephrine, norepinephrine, serotonin, bufotenine, and dihydrobufotenine) [3–6]. Bufadienolides and bufotoxins inhibit the Na^+^/K^+^ ATPase pump in the heart muscle cells. At high concentrations, these compounds may induce hallucinogenic effects by acting on the central nervous system [3–6]. It is probable that epinephrine, norepinephrine, and serotonin do not contribute significantly to the poisoning because these compounds given orally are rapidly metabolized by catechol-O-methyltransferase (COMT) in the gastrointestinal tract and by monoamine oxidase (MAO) in the gastrointestinal tract and liver [7].

The* Rhinella* genus contains more than 250 species; about 77 of them inhabit the Americas [3]. Some species with toxicological importance include* Rhinella marina* Linnaeus, 1758 (*Bufo marinus*),* Rhinella icterica* Spix, 1824 (*Bufo ictericus*),* Rhinella (Bufo) schneideri* Werner, 1894, and* Rhinella jimi* Stevaux, 2002 [3]. Undoubtedly, the most studied species is cane toad* R. marina*. The poisoning by* R. marina* toad has been reported in different species including dogs, lizards, snakes, opossums, cats, pigs, chickens, ducks, turtles, frogs, goannas, raptors, marsupial mammal, and ornamental and nonornamental fish [8–17]. Although the clinical signs induced by* R. marina* toxicity are well established in humans and mammals, its effects and pathological changes in birds are limited. This study aimed to evaluate the pathological changes that occur after administering different doses of* R. jimi* venom (Stevaux, 2002) to* Gallus gallus domesticus* chicks. This study also assessed the applicability of using chicks as experimental models in toxicity studies, which would facilitate the assessment of physiopathogenic elements and the discovery of new therapeutic approaches against this specific venom.

## 2. Materials and Methods

### 2.1. Animals

We used 23 Acoblack chicks of approximately 10 days of age bought from a local commercial producer. These animals were kept in cages (40 × 50 cm) and provided commercial poultry feed ration (Initial Ration, Purina, Sao Lourenço da Mata, PE, Brazil) and water* ad libitum*.

### 2.2. Toad Venom Collection

Four* R. jimi* toads were captured from the municipality of Limoeiro do Norte, CE, Brazil. The parotoid glands secretion was extracted by manual compression of the parotoid glands and collected in sterile bottles. Subsequently, the venom from the four toads was mixed and then suspended in distilled water to obtain the desired concentrations.

### 2.3. Experimental Design

The animals were randomly divided into 5 groups: the control group (*N* = 3), which received distilled water, and 4 groups (G3, G6, G10, and G25; *N* = 5 per group), which were treated using different concentrations of the parotoid glands secretion. The G3, G6, G10, and G25 groups received venom doses of 3, 6, 10, and 25 mg/kg, respectively. The used doses were based on earlier experimental studies [15, 18–20]. The venom was administered as a single dose by gavage. The animals were monitored for 48 h to assess any visible signs of toxicity.

After 48 h, the animals were killed by cervical dislocation and submitted for necropsy. The liver, kidney, lung, heart, spleen, bursa of Fabricius, Meckel's diverticulum, proventriculus, rectum, brain, and gizzard samples were collected and fixed in formalin. The samples were then embedded in paraffin according to routine histological processing. Sections of 5 *μ*m thickness were stained using hematoxylin and eosin (HE) for subsequent examination by using an optical microscope.

The results of scores of macroscopic pathological lesions were statistically analyzed by the chi-squared test for trend to test for linear trend across the groups. The level of significance was set at *P* < 0.05.

## 3. Results

**Figure 1:**
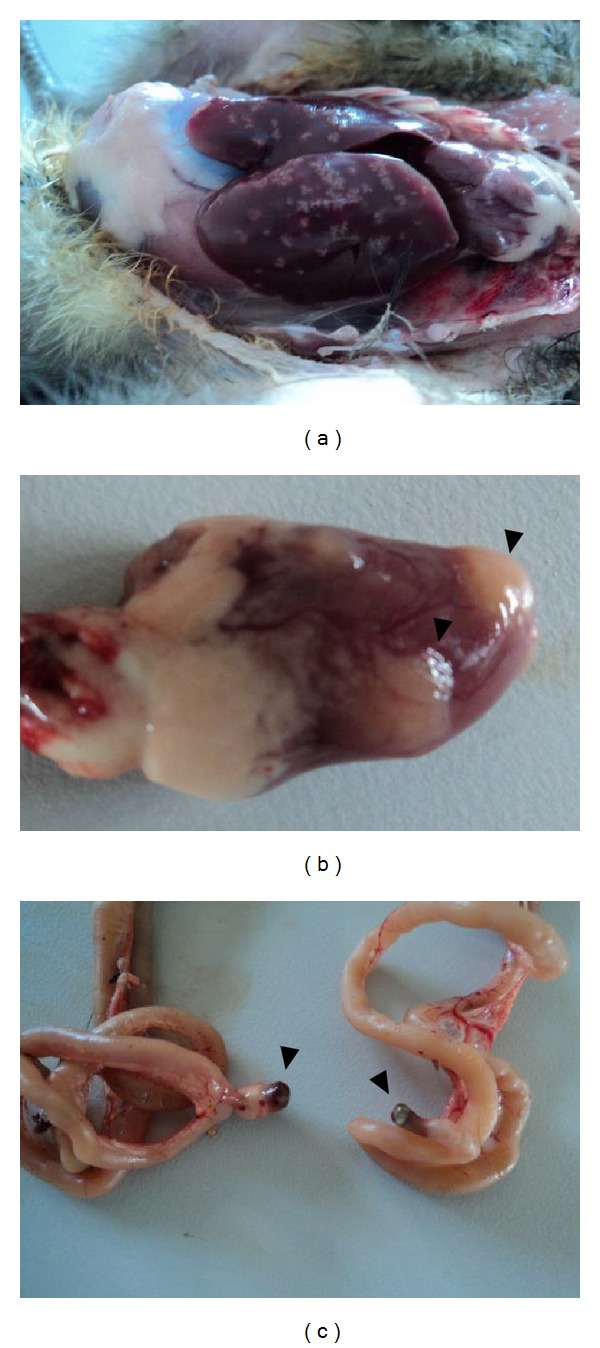
Liver (a), heart (b), and Meckel's diverticulum of chick showing suggestive areas of necrosis (arrows) after the administration of 25 mg/kg of* Rhinella jimi* venom.

No animal showed any clinical signs of toxicity. The macroscopic pathological changes observed during necropsy are presented in Table 1. No chickens in the control group showed any injury. All the animals that received the toad toxins showed hepatomegaly and multifocal yellowish white areas in the liver with sizes ranging from 1 to 3 mm, suggestive of necrosis (Figure 1(a)). The severity of the lesions appeared to be dose-dependent. Animals that received higher venom doses (10 and 25 mg/kg) showed macroscopic lesions in liver, heart, Meckel's diverticulum, spleen, lungs, and kidneys. The chi-squared test for trend of the macroscopic lesions showed significant differences for all lesions except hydropericardium.

The heart of the treated animals when observed macroscopically showed sagging, ecchymoses, focal yellowish white areas suggestive of necrosis (Figure 1(b)), and severe hydropericardium. In one animal from the G25 group, the heart showed an irregular surface with whitish nodules above the epicardium, accompanied by hemorrhagic spots. The venom-treated animals also showed splenomegaly, hemorrhagic lungs and kidneys, and dark necrotic lesions of Meckel's diverticulum (Figure 1(c)).

**Figure 2:**
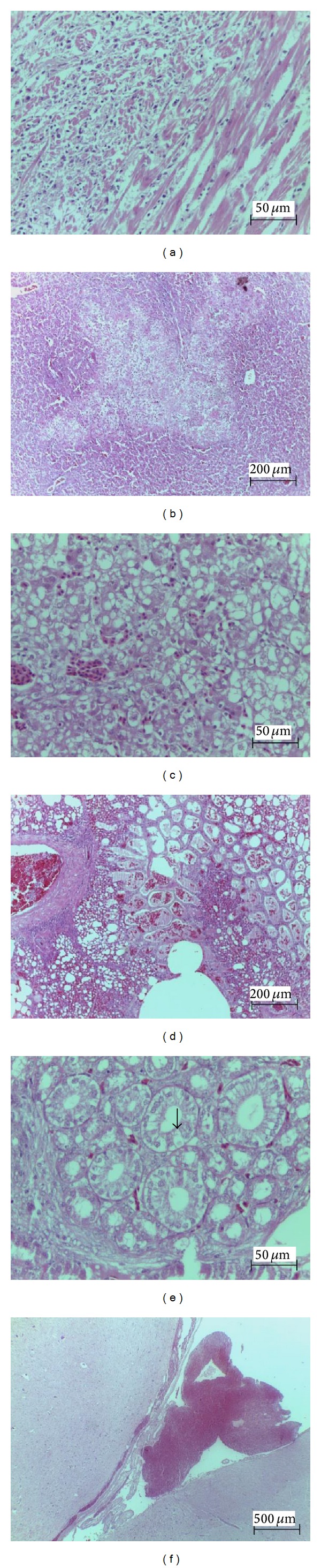
Histopathological changes in chicks treated with* Rhinella jimi *venom. (a) Extensive foci of necrotic cardiac fibers (H&E, Bar = 50 *μ*m). (b) Liver section showing hepatocyte necrosis (H&E, Bar = 200 *μ*m). (c) Focal areas of hemorrhage in the hepatic parenchyma (H&E, Bar = 50 *μ*m). (d) Lungs section showing moderate acute diffuse alveolar hemorrhage (H&E, Bar = 200 *μ*m). (e) Vacuolar degeneration of the renal tubular epithelium (arrow) and multifocal areas of hemorrhage in the interstitium of the renal cortex (H&E, Bar = 50 *μ*m). (f) Brain section showing extensive subdural hemorrhage (H&E, Bar = 200 *μ*m).

Microscopic evaluation of the treated chicks revealed lesions in the heart, liver, lungs, kidneys, and central nervous system. No significant changes were observed in the animals of the control group. The hearts of the animals from the G3 and G6 groups showed swollen cardiac fibers separated by interstitial fluid accumulation, in addition to extensive hemorrhagic foci and isolated foci of necrotic cardiac fibers, as shown by increased cytoplasmic eosinophils and the presence of pyknotic nuclei. In G10 and G25 groups, we observed extensive foci of necrotic cardiac fibers (Figure 2(a)), as well as mixed inflammatory infiltrate consisting of heterophils, macrophages, and lymphocytes between necrotic fibers.

The livers of the G3 and G6 animals showed diffuse vacuolar degeneration of hepatocytes, severe sinusoidal congestion, and focal areas of hemorrhage in the parenchyma and below the Glisson's capsule. In G10 and G25 groups, massive necrosis of hepatocytes was observed (Figure 2(b)), which was characterized by nuclear pyknosis, acidophilic cytoplasm, presence of cellular debris, and disorganized lobular architecture.

The lungs of G3 and G6 animals showed moderate acute diffuse alveolar hemorrhage (Figure 2(d)), which was characterized by a complete disruption of alveolar spaces caused by a high number of erythrocytes and the presence of variable amounts of homogeneous eosinophilic material.

The kidneys of the animals from the G3 and G6 groups showed moderate vacuolar degeneration of the renal tubular epithelium and multifocal areas of hemorrhage in the interstitium of the renal cortex. In the G10 and G25 groups, a diffuse severe vacuolar degeneration of the renal tubular epithelium was observed (Figure 2(e)). The brain samples from the G25 group showed extensive cerebral and cerebellar subdural hemorrhage (Figure 2(f)).

## 4. Discussion

In this study, the chickens that received different doses of* R. jimi* parotoid glands secretion showed no clinical signs of toxicity. Similarly, Beckmann and Shine [15] conducted a study involving chickens receiving water in which* R. marina* toads remained for 36 h, without an alternate source of water for 7 h. The study results confirmed that the chickens showed no signs of toxicity [15]. Furthermore, previous studies have shown that consuming* R. marina* tadpoles did not cause any clinical signs of toxicity in chickens [15] and domestic ducks [21]. These studies indicate that chickens and domestic ducks might be insensitive to the toxic effects of toad secretions, although no pathological studies have been conducted. However, the total bufadienolide concentrations are lower in tadpoles than in adult toads [22].

One major effect of toad toxins is cardiotoxicity [3–5, 23–25] promoted by bufadienolides, which are compounds that have a steroidal structure similar to that of digoxin [4, 26]. The lesions observed in the myocardium of humans fatally poisoned by toad toxins showed interstitial congestion and hydropic degeneration of cardiac fibers [27]. Similarly, the chicks used in this study showed extensive hemorrhagic foci and isolated foci in necrotic cardiac fibers, which represent developing pathologic cardiac lesions similar to that observed in humans.

In 5 cases of fatal human poisoning by* Bufo* spp., lung congestion and edema were observed. In 3 cases, pleural hemorrhage was detected [27]. The lungs of dogs experimentally treated with approximately 22 mg/kg of the* R. marina* crude venom showed congestion and pulmonary edema with mild perivascular mononuclear inflammatory infiltrate and moderate alveolar emphysema [19, 24]. In this study, the administration of toad venom to chickens resulted in diffuse alveolar hemorrhage in the lungs. It is possible that alterations in lung morphology were the consequence of vasoconstriction induced by biogenic amines present in the toad venom, which may be aggravated by hemodynamic changes caused by bufotoxins.

In our study, the kidneys of the treated animals showed moderate hemorrhage in the interstitium of the renal cortex and vacuolar degeneration of the renal tubular epithelium. Changes in this organ were also observed in poisoned humans, as demonstrated by kidney congestion and hydropic degeneration of the proximal tubular epithelial cells [27]. Corticomedullary congestion, mild glomerular synechiae, and presence of protein in the tubular lumen and urinary space were previously shown in toad toxins-treated dogs [19, 24]. On the basis of these findings, it may be inferred that chickens are sensitive to the nephrotoxic effects of toad toxins, similar to humans and dogs. We speculate that the hemorrhage and lesions of renal epithelial cells might be attributed to the vasoconstriction induced by biogenic amines present in the toad venom.

The chicks in our study showed lesions similar to that observed in humans and dogs. In this study, the liver of toad venom-treated animals showed vacuolar degeneration, hepatocyte necrosis, severe sinusoidal congestion, and focal areas of hemorrhage in the parenchyma and below Glisson's capsule. Humans exposed to toxins from* Bufo* spp. showed hydropic degeneration of hepatocytes [27]. Liver sections from dogs treated using* R. marina* venom showed nutmeg pattern, hepatic degeneration, multifocal congestion, and severe coagulation necrosis in the central zone of the lobules [19]. The variation in the effects may be attributable to target species differences and species-related venom composition.

Another effect observed in dogs treated with* R. marina* toxins was mild splenomegaly [19], which was also presented by chicks that received* R. jimi *venom. On the other hand, splenic congestion was observed only in humans [27]. It is possible that the toxic effects on the spleen could be because of the vasoconstriction induced by biogenic amines present in the venom.

The observed toxic effects of the toad toxins in chicks might occur in other avian species. In fact, several avian species avoid eating toad canes [13, 16] and several other species developed the ability of eating just the less toxic body parts [13]. As an interesting example, the raptors black kites (*Milvus migrans*) and whistling kites (*Haliastur sphenurus*) learned to eat just the tongues of* R. marina* probably to avoid the exposure to the toxins [28].

In conclusion, pathological changes of the* R. jimi* parotoid glands secretion in* G. gallus domesticus* chicks were noted in the heart, spleen, liver, Meckel's diverticulum, lungs, and kidneys. Most of the changes were similar to those observed in humans exposed to venom from* Bufo* spp. and in dogs treated with toxins from* R. marina*. Future research could usefully address the effects of chronic exposure of chicks to toad toxins and determine whether the preexisting diseases might increase the sensitivity.

## Figures and Tables

**Table 1:** Macroscopic pathological lesions observed in chicks treated using different doses of *Rhinella jimi* venom.

Organs	Changes	Poison dose (mg/kg)					*P* ^1^
		0 (*N* = 3)	3 (*N* = 5)	6 (*N* = 5)	10 (*N* = 5)	25 (*N* = 5)	
Liver	Hepatomegaly Necrotic areas	0 0	5 5	5 5	5 5	5 5	∗∗

Spleen	Splenomegaly	0	0	3	5	5	∗∗

Heart	Sagging Hydropericardium Hemorrhagic areas Necrosis	0 0 0 0	2 0 0 0	3 0 0 0	5 0 2 4	5 1 3 5	∗∗∗ n.s. ∗∗ ∗∗∗

Meckel's diverticulum	Dark-colored necrosis	0	0	0	4	3	∗∗

Lungs	Hemorrhage	0	0	2	4	5	∗∗∗

Kidneys	Hemorrhage	0	0	2	5	5	∗∗∗

^1^Chi-squared test for trend.

n.s.: nonsignificant; ∗: <0.05; ∗∗: <0.01; ∗∗∗: <0.001.
